# CTLA-4 Mediates Inhibitory Function of Mesenchymal Stem/Stromal Cells

**DOI:** 10.3390/ijms19082312

**Published:** 2018-08-07

**Authors:** Timo Gaber, Kerstin Schönbeck, Holger Hoff, Cam Loan Tran, Cindy Strehl, Annemarie Lang, Sarah Ohrndorf, Moritz Pfeiffenberger, Eric Röhner, Georg Matziolis, Gerd-R. Burmester, Frank Buttgereit, Paula Hoff

**Affiliations:** 1 Department of Rheumatology and Clinical Immunology, Charité–Universitätsmedizin Berlin, Humboldt-Universität zu Berlin, and Berlin Institute of Health, 10117 Berlin, Germany; kerstin.schoenbeck@charite.de (K.S.); jinluan87@yahoo.de (C.L.T.); Strehl@drfz.de (C.S.); annemarie.lang@charite.de (A.L.); sarah.ohrndorf@charite.de (S.O.); moritz.pfeiffenberger@charite.de (M.P.); Gerd.Burmester@charite.de (G.-R.B.); Frank.Buttgereit@charite.de (F.B.); 2 German Rheumatism Research Centre (DRFZ) Berlin, A Leibniz Institute, 10117 Berlin, Germany; holger.hoff@icloud.com; 3 Department of Orthopedics, Campus Eisenberg, Jena University Hospital, Klosterlausnitzer Straße 81, 07607 Eisenberg, Germany; E.Roehner@krankenhaus-eisenberg.de (E.R.); G.Matziolis@krankenhaus-eisenberg.de (G.M.); 4 Endokrinologikum Berlin, 10117 Berlin, Germany

**Keywords:** mesenchymal stem/stromal cells, immune modulation, CTLA-4, hypoxia, fracture healing, regeneration

## Abstract

Mesenchymal stem/stromal cells (MSCs) are stem cells of the connective tissue, possess a plastic phenotype, and are able to differentiate into various tissues. Besides their role in tissue regeneration, MSCs perform additional functions as a modulator or inhibitor of immune responses. Due to their pleiotropic function, MSCs have also gained therapeutic importance for the treatment of autoimmune diseases and for improving fracture healing and cartilage regeneration. However, the therapeutic/immunomodulatory mode of action of MSCs is largely unknown. Here, we describe that MSCs express the inhibitory receptor CTLA-4 (cytotoxic T lymphocyte antigen 4). We show that depending on the environmental conditions, MSCs express different isoforms of CTLA-4 with the secreted isoform (sCTLA-4) being the most abundant under hypoxic conditions. Furthermore, we demonstrate that the immunosuppressive function of MSCs is mediated mainly by the secretion of CTLA-4. These findings open new ways for treatment when tissue regeneration/fracture healing is difficult.

## 1. Introduction

The importance of tight control of the immune system is illustrated most impressively in cases when the immune system is out of control, as can be seen in cases of autoimmune diseases. In these circumstances, the untimely and almost unlimited activation of immune cells can lead to severe tissue damage that could even culminate in life-threatening conditions [1,2,3]. For keeping the immune system in check, nature has invented a variety of mechanisms that control the immune system on various levels. On the cellular level, several types of regulatory (i.e., inhibitory) cells exist, such as regulatory T (Treg) and B cells, which are able to dampen/modify or even inhibit immune responses [4,5,6]. Furthermore, several modulatory or even inhibitory mediators regulate immune responses. Prostaglandin E2 (PGE2) is able to induce the anti-inflammatory interleukin 10 (IL-10) in macrophages [7]. M2-polarized macrophages also express IL-10 but additionally express transforming growth factor β (TGFβ) and IL-10-R-antagonist [8]. CTLA-4 binding to B7.1/7.2 induces retrograde signaling within the antigen presenting cell (APC), leading to enhanced indoleamine 2,3-dioxygenase (IDO) expression [9]. 

Mesenchymal stem/stromal cells (MSCs) also exhibit immune modulatory functions and have been largely overseen as regulators. They are stem cells of the connective tissue and are most abundant in the bone marrow but can also be found (among others) in cartilage, fat tissue, blood, muscles, and liver [10,11,12,13,14]. MSCs possess a plastic phenotype and can differentiate in vitro and in vivo into different cell types, such as adipocytes and osteocytes [15,16]. Besides serving as a reservoir for new cells of mesenchymal origin, MSCs have the potential to suppress immune responses [17,18,19]. MSC-mediated immunosuppression has been addressed through several soluble mediators (such as indoleamine 2,3-dioxygenase (IDO), Haem oxygenase I, nitric oxide, IL-10, transforming growth factor β1, and prostaglandin E2 (PGE2)) but also through surface molecules (such as inhibitory molecules B7H1 and B7DC/programmed death receptor 1 (PD1) pathways) as essential effectors in blocking T cell function and inducing regulatory T cells [20,21,22].

The property of MSCs to differentiate into osteocytes and the competence of MSCs to regulate ongoing immune responses are also relevant in cases of fracture healing. Under these circumstances, MSCs are attracted to fracture hematomas where they serve as a reservoir for new osteocytes and could be responsible for down-regulation of the ongoing inflammatory phase, which is important for proper fracture healing [23,24,25,26,27,28,29]. This anti-inflammatory property of MSCs has also been exploited therapeutically by treating inflammatory diseases—such as Crohn’s disease, ulcerative colitis, and systemic lupus erythematosus—with the adoptive transfer of MSCs to patients [30,31]. However, the mechanism of action of this treatment has not been fully elucidated to date.

On the molecular level of immune regulation, cytotoxic T lymphocyte antigen 4 (CTLA-4, or CD152), a member of the CD28 family of T cell co-receptors, is an important regulator of T cell activation [32,33]. Although initially identified as a T cell-specific protein, CTLA-4 is now known to also be expressed in B cells and dendritic cells (DC) [34,35]. CTLA-4 inhibits T cell responses by several means. Having a higher affinity to bind the receptors B7.1 and B7.2, CTLA-4 out-competes the activating receptor CD28. In addition, CTLA-4 can induce DCs to switch to an inhibitory phenotype by retrograde signaling via B7.1/B7.2 on DCs. Lastly, CTLA-4 inhibits T cell activation by eliciting direct signal transduction resulting, e.g., in the activation of PI3K or the ubiquitin ligase switch [36,37,38]. The complexity of CTLA-4 function is further increased by the presence of several splice variants. Besides the full-length version of CTLA-4 (flCTLA-4) comprising the ligand-binding extracellular domain, a transmembrane domain, and an intracellular signaling domain, there is also a variant lacking the ligand-binding domain (ligand-independent CTLA-4 or liCTLA-4), another variant lacking the transmembrane domain that is secreted to the extracellular space (soluble CTLA-4 or sCTLA-4), and a short variant that lacks both the ligand-binding domain and the transmembrane domain (1/4 CTLA-4) [39]. Altered regulation of the amount of the different splice variants produced due to single nucleotide polymorphisms in the CTLA-4 gene has been found to increase the susceptibility to several autoimmune diseases [39]. 

In this study, we have analyzed the mechanism of action of MSCs to inhibit immune responses. We demonstrate that MSCs express CTLA-4 and that CTLA-4 represents the mediator of immune cell inhibition by MSCs. These findings shed new light on the possible role of sCTLA-4 for the treatment of autoimmune diseases by transfer of MSCs and open up new ways for treatment options in regeneration processes.

## 2. Results

### 2.1. MSCs Express Different Splice Variants of CTLA-4

**Figure 1:**
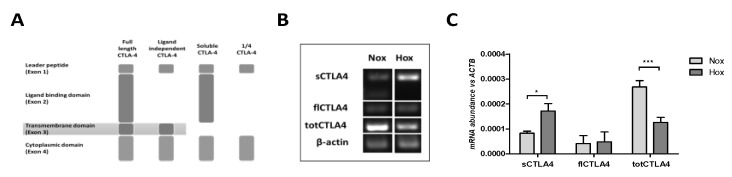
Mesenchymal stem/stromal cells (MSCs) differentially express distinct splice variants of cytotoxic T lymphocyte antigen 4 (CTLA-4) with regard to oxygen availability. (**A**) Scheme of the documented splice variants of total CTLA-4 (totCTLA-4)—full length CTLA-4 (flCTLA-4), ligand-independent CTLA-4, soluble CTLA-4 (sCTLA-4), and 1/4 CTLA-4—which were detectable in (**B**) conventional RT-PCR and (**C**) quantitative RT-PCR under normoxia (Nox; 18% O_2_) and hypoxia (Hox; 1% O_2_). Data in (**C**) are given as the mean ± SEM (statistical analysis: two-way ANOVA with Bonferroni’s *post hoc* test: * *p* < 0.05, *** *p* < 0.001).

As described above, CTLA-4 can exist in one of four splice variants: flCTLA-4, liCTLA-4, sCTLA-4, and 1/4 CTLA-4 (Figure 1A). Therefore, we analyzed the expression of total CTLA-4 comprising all isoforms as well as the expression of flCTLA-4 and sCTLA-4 individually on a transcriptional level by reverse-transcription PCR (RT-PCR). Since MSCs face bioenergetically adverse conditions, such as low oxygen levels (hypoxia) in infiltrated fracture hematomas [23], we cultivated MSCs for 72 h under either normoxic conditions (Nox, i.e., 18% O_2_) or hypoxic conditions (Hox, i.e., 1% O_2_). Most importantly, the end-point analysis of the RT-PCR showed that MSCs do express CTLA-4 on the RNA level (Figure 1B). In addition, we were able to detect the expression of both flCTLA-4 and sCTLA-4, and it appeared that sCTLA-4 is the predominant form of CTLA-4 expressed in MSCs compared to flCTLA-4. Moreover, the amount of oxygen present during cultivation of MSCs did affect the pattern of CTLA-4 splice variant expression with hypoxic conditions leading to an increase in the expression of sCTLA-4. The quantification of the CTLA-4 expression by quantitative RT-PCR (qPCR) confirmed the results of the end-point analysis and further demonstrated a generally low expression pattern as compared to the reference gene *ACTB* encoding for β-actin (Figure 1C). The qPCR revealed a higher expression of sCTLA-4 compared to flCTLA-4 and confirmed an increase in the expression of sCTLA-4 under hypoxic conditions. 

### 2.2. CTLA-4 is Expressed on the Cell Surface of MSCs

**Figure 2:**
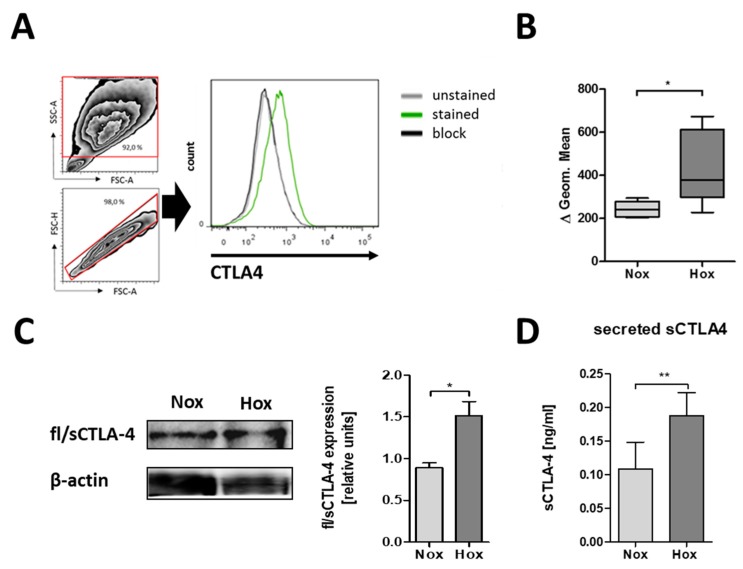
MSCs differentially express CTLA-4 protein with regard to oxygen availability. (**A**) Representative histogram of CTLA-4 surface expression on human bone marrow-derived MSCs and surface CTLA-4 staining of human MSCs as analyzed by flow cytometry. (**B**) Fluorescence intensities are depicted as box plots of delta of geometric means normalized to an antibody block (*n* = 5; * *p* < 0.05; paired *t*-test). (**C**,**D**) Intracellular CTLA-4 expression and secretion by human MSCs was confirmed by immunoblotting after native PAGE and ELISA. (**C**) Intracellular fl/sCTLA-4 (*n* = 4; * *p* < 0.05; paired *t-*test) and (**D**) secreted sCTLA-4 was enhanced in MSCs cultured under hypoxic conditions (Hox; 1% O_2_) as compared to normoxic conditions (Nox; 18% O_2_) (*n* = 6; * *p* < 0.05; paired *t*-test).

Next, we analyzed the protein expression of CTLA-4 by staining the surface of MSCs with anti-CTLA-4 antibody (Figure 2A). As a result, we observed clear CTLA-4 expression on the surface of MSCs. In addition, the cultivation of MSCs under hypoxic conditions led to a significant increase in CTLA-4 surface expression (Figure 2B). 

### 2.3. MSCs are Able to Secrete CTLA-4

The analysis of surface CTLA-4 was only able to detect flCTLA-4. To investigate whether sCTLA-4 is also expressed, we conducted native PAGE followed by immunoblots to detect intracellular CTLA-4 expression using MSC lysates. Here, we observed the expression of intracellular CTLA-4 (flCTLA-4 and sCTLA-4) being significantly induced by the reduction in oxygen levels (Figure 2C). To verify the secretion of sCTLA-4 by MSCs, we analyzed, by ELISA, the presence of CTLA-4 in the supernatants of MSCs. As shown in Figure 2D, the secreted form of CTLA-4 could be detected in the supernatants of MSCs. Additionally, cultivation of MSCs under hypoxic conditions led to a significant increase in the secretion of CTLA-4. 

### 2.4. flCTLA-4 and sCTLA-4 Mediate the Inhibitory Effect of MSCs

**Figure 3:**
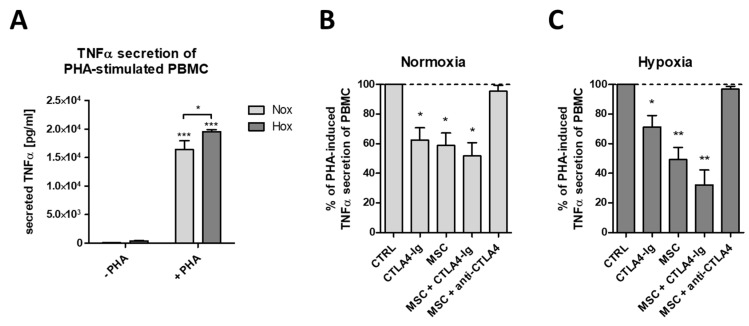
Human MSCs reduce tumor necrosis factor alpha (TNFα) secretion of mitogen-stimulated peripheral blood leukocytes in a CTLA-4-dependent manner. (**A**) Phytohaemagglutinin (PHA)-induced TNFα secretion of peripheral blood mononuclear cells (PBMCs) (*n* = 4; * *p* < 0.05; *** *p* < 0.001; two-way ANOVA with Bonferroni’s *post hoc* test). (**B**,**C**) PHA-induced TNFα secretion by PBMCs was significantly reduced by CTLA4-Ig (Abatacept), by human bone marrow-derived MSCs, and their combination (*n* = 4; * *p* < 0.05; ** *p* < 0.01; one-sample *t*-test). MSC-mediated reduction of PHA-induced TNFα secretion was blocked by anti-CTLA-4 antibody under both (**A**) normoxic (18% O_2_) and (**B**) hypoxic (1% O_2_) conditions.

To recapitulate the inhibitory effect of MSCs on the activation of T cells*,* we established an in vitro model in which tumor necrosis factor alpha (TNFα)secretion by phytohaemagglutinin (PHA)-activated peripheral blood mononuclear cells (PBMCs) is suppressed by the addition of MSCs. While PHA-activation of PBMCs led to high amounts of TNFα secretion under both normoxic and hypoxic conditions (Figure 3A), the co-cultivation of PHA-activated PBMCs with MSCs led to a significant reduction in TNFα secretion (Figure 3B,C). Interestingly, the addition of CTLA4-Ig, which blocks B7.1/B7.2, was able to mimic the inhibitory effect of MSCs on PHA-mediated PBMC activation (Figure 3B,C). A synergistic effect of MSCs and CTLA4-Ig could only be detected under hypoxic conditions (Figure 3C). Most importantly, the addition of CTLA-4 blocking antibodies to PHA-activated PBMCs and MSCs was able to abolish completely the inhibitory effect of MSCs, indicating the importance of CTLA-4 and especially sCTLA-4 for the inhibitory function of MSCs.

## 3. Discussion

In our study, we have analyzed the potential importance of CTLA-4 for the anti-inflammatory property of MSCs. To our knowledge, we are the first to provide evidence for the expression of CTLA-4 in MSCs on both an RNA and protein level and for the presence of several different splice variants in MSCs.

**Figure 4:**
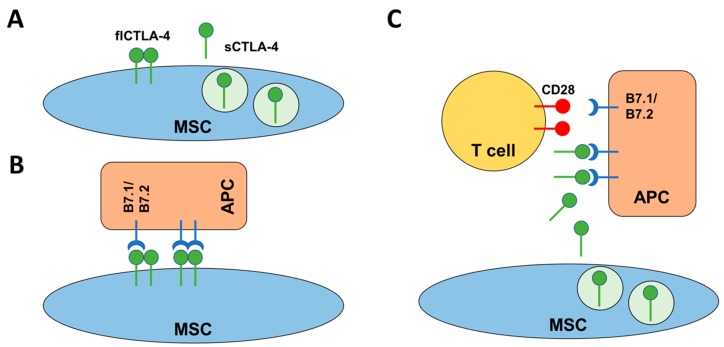
Schematic representation of the mode of action of CTLA-4 mediated inhibition of, e.g., T cell activation. (**A**) MSCs are able to express two different isoforms of CTLA-4. The flCTLA-4 is a membrane-bound dimer while sCTLA-4 is a secreted monomeric isoform of CTLA-4. (**B**) Under normoxic conditions, flCTLA-4 may mediate cell contact-dependent inhibition of T cell responses by direct interaction with B7.1/B7.2 on the surface of APCs. (**C**) Under hypoxic conditions, MSC may switch to a cell contact-independent mechanism where sCTLA-4 is able to bind B7.1/B7.2 on APCs, thereby blocking CD28 mediated co-stimulation of T cells.

The reasons for the immune privileged status of MSCs are not completely understood yet. However, several different mechanisms are currently discussed including the secretion of soluble factors and cell contact-dependent effects. Previous work suggested the expression of Fas ligand (FasL or CD95L) and IL-10 to be involved in the mechanisms by which MSCs inhibit T cell proliferation in rats [40,41]. An additional mechanism described involves the expression of indoleamine 2,3-dioxygenase (IDO) in MSCs; however, the relevance of IDO for the inhibitory effect on T cells is still under discussion [42,43]. Nevertheless, of importance is that unknown secreted factors were postulated 11 years ago [44]. Our work now adds CTLA-4 as an important cell-bound and soluble mediator of inhibitory effects to the factors already discussed (Figure 4A). We assume that MSCs not only use one mechanism of action but rather they are capable of employing different mechanisms, depending on varying environmental factors. 

Indeed, we were able to show that the expression pattern of CTLA-4 splice variants differs depending on the amount of oxygen present. This indicates that MSCs are able to adapt to different environments probably resulting in the usage of different inhibitory mechanisms. Here, we describe a scenario in which MSCs switch from a cell-contact-dependent mechanism based on flCTLA-4 under normoxic conditions to a contact-independent mechanism mediated by the secretion of sCTLA-4 under hypoxic conditions (Figure 4B,C). Our results show that both microenvironmental and bioenergetic conditions modify the behavior of MSCs. This hypothesis is summarized in Figure 4.

The transplantation of MSCs is used therapeutically to improve regenerative processes such as fracture healing and cartilage regeneration [45,46,47,48]. It has been shown that the provision of progenitor cells that differentiate into new tissues represents one mechanism by which the transplantation of MSCs positively affects regeneration [46,47,48,49,50]. Additionally, it could also be possible that it is the anti-inflammatory property of MSCs rather than the differentiation potential that positively affects at least fracture healing [24,27,28,29]. Our study shows that MSCs are able to secrete sCTLA-4 and that CTLA4-Ig is able to mimic the effect of sCTLA-4. In this regard, treatment with CTLA4-Ig could serve as an easy and cost-effective alternative to the complex and expensive cellular therapy with MSCs.

In summary, we have shown that MSCs are able to express CTLA-4, that the microenvironment of MSCs shapes the pattern of CTLA-4 expression with hypoxia resulting in the preferential expression of sCTLA-4, and that sCTLA-4 expression by MSCs can be mimicked by the addition of CTLA4-Ig. Hence, we propose that CTLA-4 is an important mediator of the anti-inflammatory effect of MSCs and that treatment of fractures with CTLA4-Ig could potentially improve fracture healing.

## 4. Material and Methods

### 4.1. Antibodies

Antibodies used for immunophenotyping of MSCs by flow cytometry were purchased from Immunotools (CD13, clone WM15, mouse IgG1, κ; CD34, clone 4H11(APG), mouse IgG1; CD45, clone MEM-28, mouse IgG1; all APC labeled), eBioscience (CD44, clone MEM-263, mouse IgG1; CD90, clone 5E10, mouse IgG1; CD105, clone SN6, mouse IgG1, κ; all APC labeled), and BD Biosciences, Heidelberg, Germany (CD73, clone AD2, mouse IgG1, κ; labeled at DRFZ using Cy5). CD14 (clone TM1, mouse IgG1) and CD19 (clone BU12, mouse IgG1), both Cy5 labeled, were obtained from the DRFZ (Berlin, Germany). Surface CTLA-4 was detected using mouse anti-human CTLA-4 (clone BNI3, mouse IgG2a, κ), purchased from BD Biosciences (Heidelberg, Germany) and labeled at DRFZ using Cy5. 

For immunoblot, mouse anti-human CTLA-4 antibody (clone BNI3, mouse IgG2a, κ) and mouse anti-human β-actin antibody (clone AC-74, mouse IgG2a) were obtained from BD Biosciences (Heidelberg, Germany) and Sigma-Aldrich Chemie GmbH, respectively.

For in vitro assays, mouse anti-human CTLA-4 (clone BNI3, mouse IgG2a, κ) and mouse isotype control IgG2a, κ were purchased from BD Biosciences, CTLA-4-Ig was obtained from Bristol-Myers Squibb GmbH & Co. KGaA (München, Germany) and corresponding control human IgG1 (Flebogamma^TM^) was obtained from Grifols (Frankfurt, Germany).

### 4.2. Isolation and Expansion and Characterization of MSCs

Human MSCs were isolated from bone marrow samples obtained from patients undergoing total hip replacement at the Center for Musculoskeletal Surgery, Charité-Universitätsmedizin Berlin. Bone marrow samples were provided by the “Tissue Harvesting” Core Facility of the BCRT ensuring the written informed consent of all human donors. All experimental protocols were approved by the ethics committee (Ethikkommission, Ethikausschuss I am Campus Charité—Mitte) of the Charité-Universitätsmedizin Berlin (EA1/012/13 from 31.01.2013) following the recommendations of the Helsinki Declaration. Human MSCs were enriched from bone marrow specimens by density gradient centrifugation and isolated via plastic adherence for 3 days whereas non-adherent cells were removed. Isolated MScs were kept under normal cultivation conditions (18% O_2_, 37 °C) in standard expansion medium (DMEM, 10% FCS, 1% Penicillin/Streptomycin) and used between passage 3 and 9. Isolation, cultivation, and characterization (surface markers CD13, CD44, CD73, CD90, CD105, CD14, CD19, CD34, and CD45 assessed by flow cytometry, Supplementary Materials Figure S1A [51], differentiation capacity described in Supplementary Materials Figure S1B) were performed according to our established standard operating procedures (SOPs). 

### 4.3. Induction of Hypoxia 

Human MSCs were incubated in a hypoxic chamber (Binder) at 5% CO_2_ and 1% O_2_ balanced with N_2_. Normoxic controls were incubated at 5% CO_2_ in a humidified atmosphere with room air oxygen content reduced by water and CO_2_ content (18% O_2_).

### 4.4. Induction of Osteogenic and Adipogenic Differentiation of MSCs

For osteogenic and adipogenic differentiation, 10,000 cells/cm^2^ were seeded in a 6-well plate and supplied with the respective conditioned medium (CM) after 24 h. Incubation was performed under standard cell culture conditions (18% O_2_, 37 °C). The medium was changed once a week and staining was performed after 28 days. Osteogenic CM included expansion medium supplemented with 10 mM β-glycerophosphate, 10 nM dexamethasone, and 0.1 mM l-ascorbic acid-2-phosphate. The adipogenic CM was supplemented with 10 µg/mL insulin, 0.2 mM indomethacin, 1 µM dexamethasone, and 0.5 mM 3-isobutyl-1-methylxanthine. 

### 4.5. Oil Red O Staining of Adipogenic Differentiated MSCs

Adipogenic differentiation is characterized by the formation of droplet accumulation that can be visualized via Oil Red O staining. Therefore, cells were fixed with 4% paraformaldehyde for 20 min at room temperature and then washed with distilled water and 60% isopropanol. Oil Red O stock solution (0.5 g Oil Red O in 100 mL 100% isopropanol) was diluted and filtered through a 0.25 µm syringe filter after 10 min incubation. The 60% working solution was applied to the cells and incubated for 15 min. After washing (60% isopropanol, distilled water), imaging was performed by light microscopy.

### 4.6. Alizarin S Red Staining of Osteogenic Differentiated MSCs

Cells undergoing osteogenic differentiation were stained with Alizarin S Red dye (Sigma Aldrich, Hamburg, Germany) to visualize calcium deposits. To this end, the medium was removed and the cells were fixed with 4% paraformaldehyde (20 min) and stained with Alizarin S Red (Sigma Aldrich) (10 min). Imaging was performed at room temperature by microscopy.

### 4.7. RNA Isolation and Quantitative PCR (qPCR) of CTLA-4 Genes of MSCs

Total RNA was extracted using Arcturus™ PicoPure™ RNA Isolation Kit (Applied Biosystems, Darmstadt, Germany) according to the manufacturer’s instructions and the RNA concentration was determined using Nanodrop ND-1000 (Peqlab Biotechnologie, Erlangen, Germany). RNA was stored at −80 °C until further processing. The cDNA was synthesized by reverse transcription using TaqMan^®^ Reverse Transcription Reagents (Applied Biosystems) for RNA concentrations >50 ng/µL or Sensiscript^®^ Reverse Transcription Kit (QIAGEN GmbH, Hilden, Germany) for RNA concentrations ≤50 ng/µL. cDNA was stored at −20 °C until further processing. qPCR was carried out using the LightCycler^®^ Fast Start DNA Master SYBR^®^ Green I Kit (ROCHE Diagnostics-Applied Science, Mannheim, Germany) according to the following cycle program: Initial denaturation was for 10 min at 95 °C followed by 50 cycles with 5 s at 98 °C, 7 s at 64 °C, and 9 s at 72 °C. Finally, the melting curve was analyzed by increasing the temperature stepwise from 60 °C to 95 °C every 30 s. Data were acquired using the Stratagene Mx3000P (Agilent Technologies, Santa Clara, CA, USA) and normalized to the expression of β-actin (*ACTB*) using the ΔCt method. All primers used were obtained from TIB Molbiol (Berlin, Germany) and are summarized in Table 1.

### 4.8. Immunoblot of CTLA-4 and β-Actin

For analyzing CTLA-4 expression in MSC, 10^6^ cells were seeded and harvested after 72 h of incubation under either normoxia or hypoxia. Cell lysis was performed in 20 μL ddH_2_O after repetitive freeze-thaw cycles (four times) using liquid nitrogen. Whole protein extracts were separated by native PAGE and blotted onto PVDF membranes (Millipore, Billerica, MA, USA). Blotted proteins were analyzed as indicated and visualized by enzymatic chemiluminescence (Amersham Biosciences, Freiburg, Germany). Image analysis was performed using ImageJ v.1.52a (http://imagej.nih.gov/ij, Wayne Rasband, National Institutes of Health, USA).

### 4.9. Quantification of Secreted CTLA-4

For quantifying secreted CTLA-4, cell culture supernatants from MSC (10^6^ cells seeded on a 6-well in 3 mL fully supplemented DMEM) were incubated for 72 h under either normoxic or hypoxic conditions, harvested, and stored at −70 °C. Secreted CTLA-4 was quantified by LEGEND MAX™ Human Soluble CTLA-4 ELISA Kit with pre-coated plates according to the manufacturer’s instructions (BioLegend^®^, San Diego, CA, USA). The OD was measured at 450 nm. A standard curve was generated by four-parametric logistic curve fit.

### 4.10. Quantification of Secreted TNFα

For determining the impact of CTLA-4 on TNFα secretion, pre-activated human PBMCs were co-cultured with pre-treated MSC for 48 h. To this end, human PBMCs were isolated by density gradient centrifugation using Ficoll-Paque™ PLUS (GE Healthcare, München, Germany). PBMCs (0.5 × 10^6^/mL) were stimulated for 48 h using PHA-L (5 µg/mL) whereas MSCs (0.5 × 10^6^/mL) were pre-treated with either 2 µg/mL control mouse IgG or 2 µg/mL mouse anti-CTLA4-IgG (both BD Biosciences). Pre-stimulated PBMCs were treated with either 2 µg/mL control human IgG (Grifols) indicated as MSC or 2 µg/mL humanized CTLA4-Ig (Bristol-Myers Squibb) and added in a 2 to 1 ratio to the pre-treated MSCs (indicated as MSC or MSC + CTLA4-Ig and MSC + anti-CTLA-4) or to the pre-treatment conditions without MSC (indicated as CTRL or CTLA4-Ig). 

Cell culture supernatants were harvested for quantification of TNF secretion and stored at −70 °C until analysis. TNFα DuoSet^®^ ELISA was performed according to the manufacturer’s instructions (R&D Systems, Wiesbaden, Germany). The OD was measured at 450 nm and 540 nm. A standard curve was generated with a four-parametric logistic curve fit.

### 4.11. Statistical Analysis

Data shown are reported as the mean ± SEM of at least three independently performed experiments. Differences between normally distributed groups were compared using the student′s *t* test. Multiple comparisons were analyzed by two-way ANOVA as indicated with Bonferroni's multiple comparison *post hoc* test. Statistical significance was considered when *p* < 0.05.

## Figures and Tables

**Table 1:** qPCR primer sets.

Symbol	Gene Name	Accession Number	Forward Primer	Reverse Primer	Remarks
*flCTLA4*	Homo sapiens cytotoxic T-lymphocyte associated protein 4 (*CTLA4*), transcript variant 1, mRNA	NM_005214	*ACC CAG ATT TAT GTA ATT GAT CCA GAA*	*CCG AAC TAA CTG CAA GGA*	Primer combination detects transcript variant 1, which represents the longer transcript and encodes the longer membrane-bound isoform CTLA4-TM (also known as full-length (fl)CTLA4)
*sCTLA4*	Homo sapiens cytotoxic T-lymphocyte associated protein 4 (*CTLA4*), transcript variant 2, mRNA	NM_001037631	*ATG TAA TTG CTA AAG AAA AGA AGC CCT C*	*GCCTCAGCTCTTGGAAATTGAAAT*	Primer combination detects the encoded isoform CTLA-4delTM (also known as sCTLA4) which is soluble and lacks the transmembrane domain, compared to isoform
*totCTLA4*	Homo sapiens cytotoxic T-lymphocyte associated protein 4 (*CTLA4*), transcript variant 1 and 2, mRNA	NM_005214 and NM_001037631	*TGT TGA CAT GTG CTT TGG GG*	*GCT GCC TTC TGT CCA TG*	Primer combination detects all transcript variants of CTLA4 including flCTLA-4 and sCTLA-4 but also the ligand independent CTLA-4 and the 1/4 CTLA-4 that lacks both the ligand-binding domain and the transmembrane domain
*ACTB*	Homo sapiens actin beta (*ACTB*), mRNA	NM_001101	*GAC AGG ATG CAG AAG GAG ATC ACT*	*TGA TCC ACA TCT GCT GGA AGG T*	Primer combination detects full length ACTB

## Supplementary Materials

Supplementary materials can be found at http://www.mdpi.com/1422-0067/19/8/2312/s1.
