# Supplementary material for: CTLA-4 Mediates Inhibitory Function of Mesenchymal Stem/Stromal Cells
Source: Int J Mol Sci. 2018 Aug 7;19(8):2312. doi: 10.3390/ijms19082312 (PMC6121442; doi:10.3390/ijms19082312)
Supplement: Supplementary file 1 [file ijms-19-02312-s001.pdf]

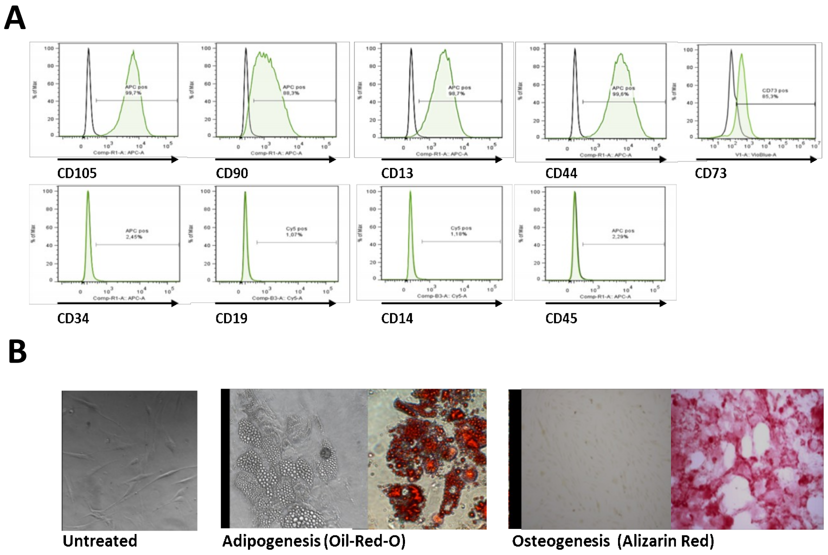

Supplemental Figure 1. Human bone marrow-derived MSCs are characterized by specific markers and differentiation capacity. The plastic adherent MSCs are characterized (**A**) by the expression of surface marker CD13, CD44, CD73, CD90 and CD105, but the lack of CD14, CD19, CD34 expression and CD45 and (**B**) by their differentiation capacity towards adipogenesis and osteogenesis as demonstrated by Oil-Red-O staining and Alizarin Red staining, respectively.
